# Nanocomposites Polarizing by Absorption: Dichroism in the Near-Infrared Region (NIR)

**DOI:** 10.3390/ma7031899

**Published:** 2014-03-05

**Authors:** Lorenz Bonderer, Dirk I. Uhlenhaut, Paul Smith, Walter Caseri

**Affiliations:** Department of Materials, Eidgenössische Technische Hochschule (ETH) Zürich, Wolfgang-Pauli-Strasse 10, Zürich CH-8093, Switzerland; E-Mails: lorenz@bonderer.ch (L.B.); dirk.uhlenhaut@alumni.ethz.ch (D.I.U.); paul.smith@mat.ethz.ch (P.S.)

**Keywords:** nanocomposites, polymers, gold nanoparticles, alkanethiols, surface modification, elastomers, poly(dimethylsiloxane), optical properties, dichroism, near infrared

## Abstract

We describe the preparation of nanocomposites which exhibit dichroism in the near infrared region (NIR). These materials consist of crosslinked poly(dimethylsiloxane) (PDMS) and gold nanoparticles, coated with 1-dodecanethiol or *tert*-tetradecanethiol. The alkanethiols improve dispersibility of the gold particles, and accordingly composites were manufactured by diffusion of the particles into swollen self-supporting PDMS elastomer films. After drying, the films were exposed to solvents for one minute, stretched in wet state, dried again and annealed. This procedure led to formation of oriented linear gold particle assemblies within stretched polymer. If the aspect ratio of the particle assemblies is high, the absorption of polarized light in the NIR region is expected to depend on the angle between the polarization plane and the orientation direction of the particle assemblies, and this was observed to be the case.

## 1. Introduction

A number of nanocomposites based on natural or synthetic polymers and isotropic inorganic nanoparticles, in particular gold or silver, have been found to show dichroic behavior, *i.e*., the interaction of polarized light with those materials depends on the angle between a specified sample axis and the polarization plane of the light. Because this phenomenon has been manifest in the visible wavelength regions, the color of films viewed through a polarizer changed when the polarizer was rotated. Such nanocomposites attracted attention first in biology [1–7] and subsequently also in materials science [8–16]. It was recognized early [4,7,17,18] that the dichroism originates in uniaxially oriented, linear assemblies of particles, whereas the interaction of these arrays with polarized light depends on the angle of the polarization plane of the incident light and the orientation axis of the particle assemblies. In addition, it was described that the dichroic effect of oriented linear particle assemblies is similar to rod-like nanoparticles oriented in a polymer matrix [19–22].

Dichroic nanocomposites have been prepared typically by reduction of metal ions in solid natural or synthetic polymer matrices [1–3,9,13–15], or based on mixing (surface-modified) metal particles with polymers in the molten [11,23] or dissolved state [10–12,20,21]. In the case of polymer solutions containing dispersed nanoparticles, nanocomposite films were obtained by casting and solvent evaporation, sometimes followed by hot pressing to improve the homogeneity of the samples [10–12,15]. Orientation of nanoparticle assemblies was finally achieved by gathering of particles in oriented elongated cavities of the matrix [4,7,9,17,18], by drawing of the polymers [1,3,10–12,14,15,19,21] or by melt elongation techniques [23].

**Figure 1.**
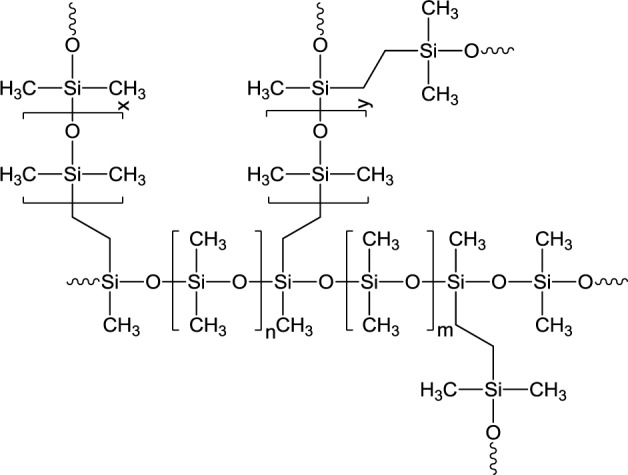
Schematic representation of crosslinked poly(dimethlsiloxane) (PDMS).

In all these examples dichroism was essentially observed in the region of visible wavelengths. Although UV-vis spectra indicated that predominance of dichroism might be shifted to the near-infrared (NIR) region [16], this possibility has so far, to our knowledge, not been substantiated. Herein, we therefore describe the preparation of nanocomposites which exhibit a dichroism dominating the near-infrared (NIR) region. This was achieved with a new preparation method, which is based on the diffusion of alkanethiol-modified particles into the swollen crosslinked elastomer poly(dimethylsiloxane) (PDMS) (Figure 1).

## 2. Results and Discussion

### 2.1. Alkanethiol-Modified Gold Nanoparticles

**Figure 2.**
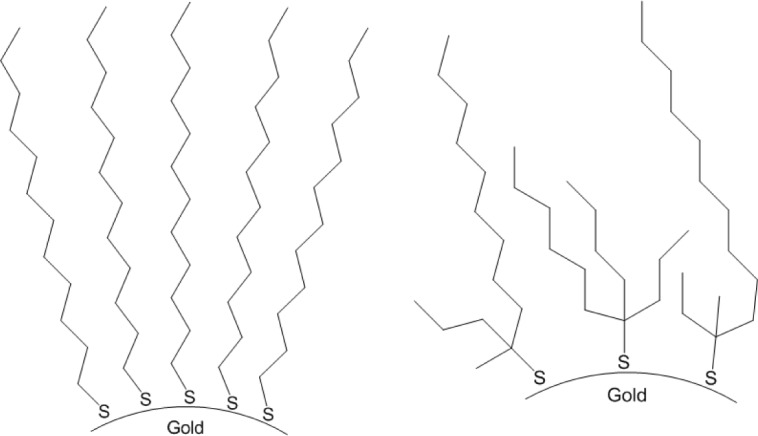
Schematic representation of gold nanoparticles covered with 1-dodecanthiol or *tert*-tetradecanethiol, respectively, assuming adsorption as thiolates.

In order to obtain materials with dichroism in the NIR region, the employed gold particles were coated with a layer of 1-dodcanethiol or *tert*-tetradecanethiol, the latter designating HSC{(CH_2_)*_x_*CH_3_}{(CH_2_)*_y_*CH_3_}{(CH_2_)*_z_*CH_3_} (*x* + *y* + *z* = 10) (we refer to alkanethiol layers although these agents may convert to thiolates or disulfides upon adsorption) (Figure 2). 1-Dodecanethiol is a convenient agent for modification of gold nanoparticle surfaces, while *tert*-tetradecanethiol was selected because it was thought that the branched and irregular composition of the alkyl groups might promote dispersibility of the particles coated therewith.

**Figure 3.**
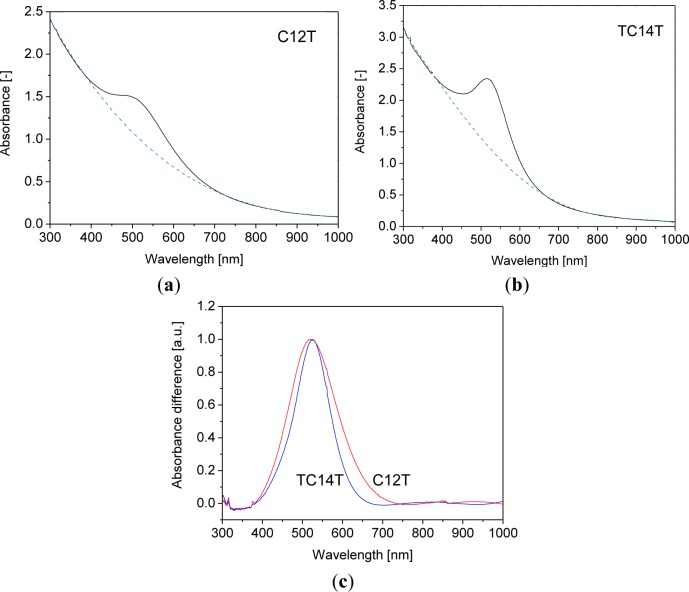
UV-vis spectra of gold nanoparticles coated (**a**) with 1-dodecanethiol (C12T) or (**b**) *tert*-tetradecanethiol (TC14T); dispersed in toluene (concentration *ca*. 0.2 mg/mL). The dashed line represents the absorbance of an arbitrarily fitted background on the basis of a 4th order polynomial; (**c**) absorbance of the alkanethiol-coated particles after subtraction of the 4th order background, normalized to 1 to the peak maximum.

**Figure 4.**
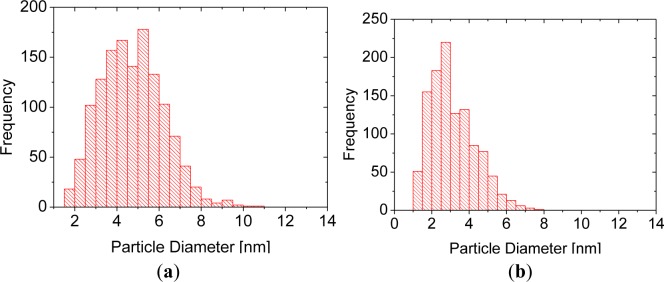
Particle size distribution of gold nanoparticles covered (**a**) with 1-dodecanthiol or (**b**) *tert*-tetradecanethiol (right panel), respectively, from transmission electron microscopy (TEM).

The particles were synthesized following procedures described in the literature, based on the reduction of tetrachloroaurate(III), in a two phase system consisting of water and toluene, with dissolved alkanethiol in presence of a phase transfer catalyst [24] (for details and chemical analyses see Experimental section). This method yields particles with average core diameters in the range of 2–5 nm [12,24], which we have controlled for *tert*-tetradecanethiol- and 1-dodecanethiol- capped gold particles (3 nm and 5 nm, respectively, according to transmission electron microscopy). Accordingly, the vis spectra of the particles dispersed in toluene showed an absorption maximum at 520–525 nm after subtraction of the 4th order background (characteristic for scattering, Figure 3), as expected for the surface plasmon resonance of alkanethiol-coated gold particles with core diameters in the indicated size range [25]. In case of pronounced agglomeration, a shift of the absorption maximum towards higher wavelengths would have been expected [16]. The surface plasmon band of the *tert*-tetradecanethiol-capped particles is somewhat narrower than that of the 1-dodecane-capped particles, which could be due to differences in surface coverage of alkanethiol or to distribution of the primary particle diameters. Indeed, the particle size distribution, evaluated by transmission electron microscopy (TEM), of the *tert*-tetradecanethiol-capped particles is somewhat narrower than for the 1-dodecanethiol-capped particles (Figure 4).

For freshly prepared dispersions of 1-dodcanethiol-coated gold particles in toluene and *tert*-tetradecanethiol-coated gold particles in toluene or heptane, the law of Bouguer-Lambert-Beer [26–30] was found to be valid in a concentration range of 0.01%–0.20% w/w, *i.e*., the absorbance in optical spectra (around 500 nm) was proportional to the concentration of the particles. However, the absorbance around 500 nm increased up to 20% within 2.5–3 days, for reasons as yet unknown.

### 2.2. Preparation of Polydimethylsiloxane (PDMS) Films Comprising Gold Nanoparticles

In order to incorporate gold nanoparticles into PDMS, swollen crosslinked PDMS films were exposed to dispersions of *tert*-tetradecanethiol- or 1-doceanethiol-capped particles. The PDMS matrix was synthesized by copolymerization of two PDMS components modified with a small fraction of vinyl- or hydrosilane moieties, respectively. When such elastomers were exposed to organic solvents, swelling proceeded readily. For instance, upon treatment of pristine films (*i.e*., films without gold particles) to toluene or heptane, the mass of the films increased by roughly 8–11 fold of the original mass within 0.5–1 h. In the following, toluene was selected as the solvent as it yielded visually clear dispersions of both *tert*-tetradecanethiol- and 1-doceanethiol-capped particles while only the former particles dispersed well in heptane.

Crosslinked PDMS films were exposed to homogeneous dispersions of *tert*-tetradecanethiol- or 1-dodcanethiol-coated gold particles for seven days. Investigation of the dispersions by UV/vis spectroscopy was performed periodically with the aim to identify the time required for reaching the equilibrium state of diffusion, yet the above mentioned temporal changes in the absorbances of alkanethiol-coated gold particles upon prolonged time rendered quantitative evaluations problematic. It was, however, qualitatively evident that gold particles diffused into the PDMS matrix rapidly upon swelling in toluene, and probably the major fraction of the particles was incorporated in the swollen PDMS matrix already within about one hour.

When the samples were removed from the particle dispersions after 7 days and subsequently dried at ambient conditions, films were obtained with typical thicknesses of around 0.5 mm. The gold content of representative films in the dried state was analyzed by atomic absorption spectroscopy. After immersion of PDMS in toluene dispersions containing 0.2% w/w 1-dodecanethiol-coated particles (this fraction includes the alkanethiol layer), a gold content of 0.39% w/w was found in the dried films. This quantity increased to 1.27% when a 0.6% w/w dispersion was applied, and a similar value (1.34% w/w) resulted when 0.6% w/w dispersions of *tert*-tetradecanethiol-capped gold particles were used.

### 2.3. Stretching of PDMS-Gold Films, Dichroism in the NIR Region

As mentioned in the Introduction, drawing of isotropic polymer films comprising gold or silver nanoparticles has been used previously as a method for the preparation of nanocomposites with dichroism in the region of visible wavelengths. Hence, drawing was also applied for the above described PDMS-gold nanocomposites. Because PDMS is an elastomer, a manually operated stretching tool was used to allow controlled stretching of clamped samples. When dried samples comprising 1-dodcanethiol- or *tert*-tetradecanethiol-coated gold particles were stretched by a factor of 3–4 and inserted in the stretched state (*i.e*., together with the stretching tool) in a vis-NIR spectrometer, no noteworthy dichroism in the vis-NIR region was observed. Also, when dried PDMS-gold films were immersed in dichloromethane or toluene for 60 s, stretched in the wet state, dried at ambient conditions and examined with vis-NIR spectroscopy in the stretched state, at best a slight dichroism was found in the region of visible and near infrared wavelengths.

**Figure 5.**
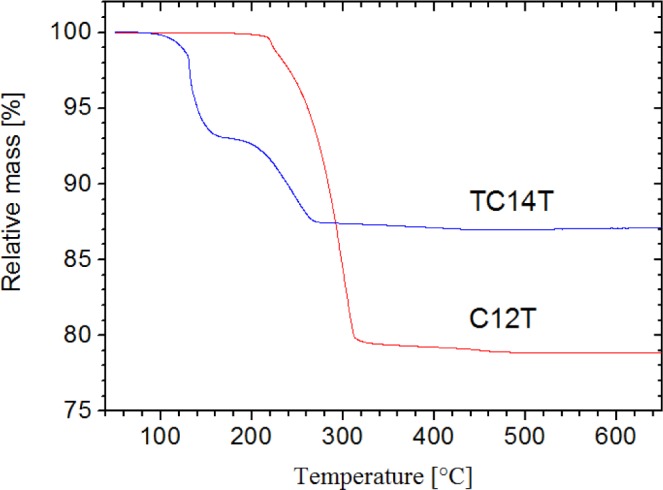
Thermogravimetric analysis of gold particles coated with 1-dodecanethiol (C12T) or *tert*-tetradecanethiol (TC14T).

However, suitable conditions for providing samples with a dichroism dominating the NIR region were established by immersion of dried films in dichloromethane or toluene for 60 s, stretching by a factor of 3–4 in the wet state, drying in ambient atmosphere and annealing in the stretched state at 120 °C, in the case of particles covered with *tert*-tetradecanethiol, or 220 °C with 1-dodecanethiol. Preliminary experiments suggested that there is an optimum annealing time, *i.e.,* too short or too long periods did not lead to significant dichroism. Also, films which were not stretched did not become dichroic upon thermal treatment. Not unexpectedly, dichroism was more distinct in samples with about 1.3% w/w than with 0.4% w/w particles. Thermogravimetric analyses imply that desorption of alkanethiols proceeds at the annealing temperature (Figure 5, the higher thermal stability of the 1-dodcanethiol-capped particles is probably due to denser packing of linear alkanethiols). It has been shown previously that even partial desorption of alkanethiols from gold can give rise to formation of particle assemblies [12].

**Figure 6.**
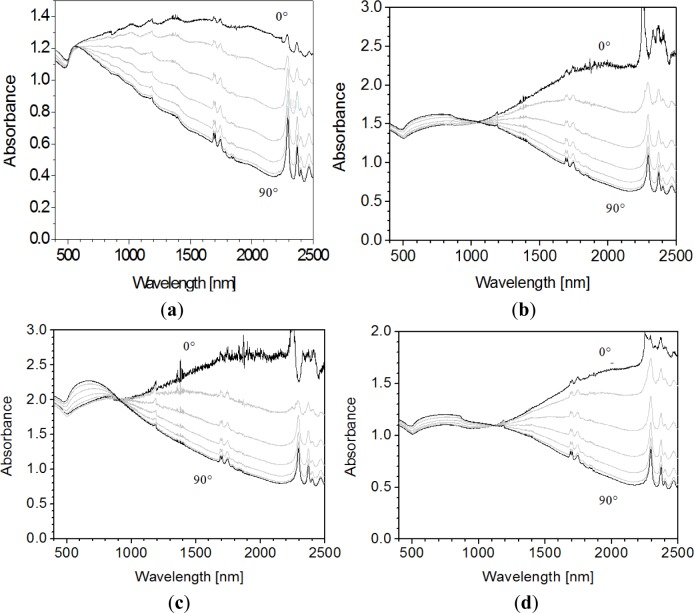
Vis-NIR spectra in polarized light of crosslinked PDMS films containing gold nanoparticles coated with 1-dodecanthiol (C12T) or *tert*-tetradecanethiol (TC14T) (φ = angle between the polarization plane of the light and the stretching direction of the films, in steps of 15°). The films were stretched after immersion in toluene for 60 s, subsequently dried in air in the stretched state and further treated as follows: (**a**) C12T, stretching factor (*SF*) = 3, relaxed to its original size, placed between two polyimide foils and inserted in this configuration between two hot metal plates at 220 °C for 10 min at more or less ambient pressure (spectrum in the relaxed state); (**b**) TC14T, *SF* = 3, heated and annealed at 120 °C for 40 min in the stretched state (spectrum also in the stretched state); (**c**) similar to (**b**) but *SF* = 4; (**d**) similar to (**b**) but subsequently stretched in the dry state to a final SR of 4.5.

Figure 6 shows a selection of vis-NIR spectra recorded with polarized light of samples annealed under different conditions and stretched at different ratios (1.3% w/w gold). They showed the same essential features: all spectra were dominated by a marked dichroism in the NIR region (a less pronounced dichroism also arose in the visible wavelength region), and a distinct isosbestic point emerged below 1200 nm. At parallel position of polarization plane of incident light and stretching direction (*i.e*., the angle φ between polarization direction of the light and stretching direction is 0°), the absorption maximum was extremely broad and emerged between 1500 nm and 2200 nm. At perpendicular orientation (φ = 90°) absorption maxima typically arose at 600–800 nm (occasionally somewhat lower values such as 570 nm were found, *cf*. Figure 6a), and the absorbance at φ = 90° decreased continuously in the NIR region. In the case of gold nanorods, the transverse resonance emerges near the plasmon resonance of a single gold particle with similar diameter [31]. However, the plasmon resonances of single spheric gold particles arise well below 600 nm; also for large nanoparticles (diameters above 100 nm) [32]. Thus, the diameter of a particle with an absorption maximum of 700 nm should be enormous and its length in a range accessible to optical microscopy, which, however, was not the case. A possible explanation for this deviation could originate in baseline effects. Note that, in particular, the transverse bands with maxima at higher wavelengths are very broad and, in addition, only relatively slightly above the absorption of the longitudinal absorption band (Figure 6b,d). Therefore, relatively small deviations in the baselines of the spectra can cause pronounced shifts of the absorption maxima in the wavelength region of the transverse absorption band, as also indicated by Figure 3.

Both the absorption maxima at φ = 0° and φ = 90° were shifted towards higher wavelengths than commonly reported for dichroic nanocomposites comprising gold or silver particles [10–16,19–23], and the difference between the absorption maxima at φ = 0° and φ = 90° was larger in the materials presented here. The sharper signals superimposed on the broad absorption bands in the NIR region are attributed to vibrations of bonds in PDMS, which has been reported to show numerous distinct peaks in the NIR region [33–35].

It is established that dichroism in polymer-nanoparticle systems originates in uniaxially oriented linear particle assemblies [10–12]. The interaction of linear particle assemblies with linearly polarized light depends on the angle between the polarization plane of light and the orientation axis of the particle assemblies, similar to rod-like gold nanoparticles [36]. Hence, as for gold nanorods of high aspect ratio [37], the absorption maximum wavelength (λ_max_) at φ = 0° is expected to shift into the NIR region. Therefore the PDMS-gold nanocomposites described here are likely to contain linear particle assemblies of high aspect ratio, with the long axis oriented parallel to the stretching direction (at perpendicular direction, λ_max_ would be expected to be higher at φ = 90° than at φ = 0°). Initially, the particles exhibit certain mobility in the presence of a solvent, so they might assemble in the anisotropic cavities of the elongated matrix—since stretching in the dry state did not lead to a pronounced dichroism, some mobility of the particles appears to be of importance to generate dichroism. In addition, some desorption of the alkanethiol layer around the gold particles, and the heat treatment, seem to be essential. This could indicate contact between gold particles in the oriented hollow spaces upon (partial) desorption of the alkanethiol layer, thus leading to closer packed gold particle assemblies. Based on the position of longitudinal absorption maxima of gold nanorods [31], the aspect ratio of the particle assemblies in the elongated PDMS matrices might fall in the range of 10–20. Therefore, if the diameter of the assemblies corresponded to about 10 nm, as is the case of gold particles in elongated polyethylene [12], the length of the assemblies might amount to about 100–200 nm, *i.e*., the order of magnitude of the number of particles in cylindrically shaped assemblies might amount to 200–500.

## 3. Experimental Section

### 3.1. Materials and Method

Poly(dimethylsiloxane-*co*-methylvinylsiloxane) (VDT-153, 0.3%–0.7% mol/mol methylvinylsiloxane, viscosity 2 × 10^5^–4 × 10^5^ cSt (1 cSt = 10^−6^ m^2^·s^−1^)) and poly(dimethylsiloxane-*co*-hydromethylsiloxane) (HMS-013, 0.5%–1.0% mol/mol hydromethylsiloxane, viscosity 6 × 10^3^–8 × 10^3^ cSt) were purchased from ABCR (Karlsruhe, Germany), sodium tetrachloroaurate from Alfa Products/Johnson Matthey GmbH (Karlsruhe, Germany), tetraoctylammonium bromide and sodium borohydride from Fluka Chemie AG (Buchs, Switzerland), and 1-dodecanethiol and *tert*-tetradecanethiol (mixture of isomers) from TCI Europe (Zwijndrecht, The Netherlands).

The mass fraction of C and H in the alkanethiol-coated particles was measured by the Microelemental Service of the Laboratory of Organic Chemistry at ETH Zürich. Gold analyses were performed by Max Steidle from Peter Link AG, Ebnat-Kappel, Switzerland. Hereby, the samples were heated in a solution of sulfuric acid and perhydrol (hydrogen peroxide) until the reaction mixture consisted of a clear solution. Thereafter, the solvents were completely evaporated, the remaining residues were dissolved in aqua regia, and after dilution with water the gold contents were determined by flame atomic absorption spectroscopy.

Thermogravimetric analyses (TGA) were performed on a Perkin Elmer TGA7 instrument under nitrogen atmosphere at heating rates of 20 K/min. Vis-NIR measurements were carried out with a Perkin Elmer Lambda 900 UV/VIS/NIR double beam spectrometer, using Suprasil quartz glass cuvettes of 1 cm path length for measurements of dispersions. Nanocomposites were investigated as free standing films or clamped in a manually operated tensile device within which the various films were deformed.

### 3.2. Synthesis and Analysis of Alkanethiol-Modified Gold Particle

The synthesis of alkanethiol-coated gold particles was essentially based on the literature [24], but was slightly modified. Accordingly, 150 mL of an aqueous solution of sodium tetrachloroaurate (30 mmol/L) (NaAuCl_4_) was mixed with 400 mL of a toluene solution of tetraoctylammonium bromide (50 mmol/L) and vigorously stirred for 60 min. The solution was protected from light. Then 4.2 mmol of the respective alkanethiols (1-dodecanethiol or *tert*-tetradecanethiol) were added, and 125 mL of a freshly prepared aqueous solution of sodium borohydride (0.4 mol/L) was dropped into the mixture under stirring during 60 min. Thereafter the resulting mixture was stirred for another 180 min. The dark red toluene phase was separated from the clear and transparent water phase with a separatory funnel and evaporated to approximately 10 mL by means of a rotary evaporator (*T* = 310 K, *p* < 75 mbar). The remaining viscous liquid was mixed with 1.5 L ethanol to remove excess alkanethiol and phase transfer agent. This mixture containing 1-dodecanethiol was cooled in a sodium chloride/ice mixture at 250–270 K overnight to precipitate the surface-modified nanoparticles, which then were filtered, re-dispersed in 50 mL toluene to yield a clear mixture and precipitated again by addition of 1 L ethanol. In the case of the *tert*-tetradecanethiol covered particles, precipitation occurred in a dry-ice/isopropanol bath overnight at a temperature of 195 K. Complete precipitation was not achieved, as evident from the fact that the outstanding solution was still colored. The filtered and dried (about 10 mbar, overnight) products were of a waxy, sometimes tacky consistence and of a dark red colour. The yield amounted to 726 mg for the 1-dodecanethiol-coated particles and 551 mg for the tetradecanethiol-coated particles. Elemental analysis revealed 15.50% w/w C and 2.74% w/w H for the 1-dodecanethiol-coated particles and 9.64% w/w C and 1.70% w/w H for the *tert*-tetradecanethiol-coated particles. For the latter, the gold content was also analysed and amounted to 85% w/w, corresponding essentially to the residual mass of 87% w/w in TGA at 1173 K (79% w/w for 1-dodecanthiol-coated particles). Most likely, the bulky structure of the *tert*-tetradecanethiol group inhibited a dense packing of the sulfur atoms onto the particle surfaces.

### 3.3. Preparation of Nanocomposite Films

Crosslinked poly(dimethylsiloxane) (PDMS) elastomer films were prepared by catalytic hydrosilylation of two PDMS components containing either Si-H or olefin groups. Accordingly, 12.0 g poly(dimethylsiloxane-*co*-methylvinylsiloxane) were dissolved in 100 mL toluene under stirring for several hours. In another vessel, 20.9 mg *cis*-(PtCl_2_(styrene)_2_) (prepared according to the literature [38]) was dissolved in 50 g toluene by stirring for several hours. Thereafter, 363.5 mg poly(dimethylsiloxane-*co*-hydromethylsiloxane) were placed in a 100 mL glass beaker, and 23.0 g of the poly(dimethylsiloxane-*co*-methylvinylsiloxane) solution and 2.00 g of the *cis*-[PtCl_2_(styrene)_2_] solution were added. The resulting mixture was stirred for 3–5 min and then instantly poured into a Teflon dish with an inner diameter of 9 cm. The dish was covered with porous soft tissue in order to prevent contamination of the reaction mixture by ambient dust, and the solvent was allowed to evaporate during several days at ambient conditions.

For diffusion of surface-modified gold particles into crosslinked PDMS, surface-modified gold particles were dispersed in toluene (particle concentrations indicated in the text, typically 0.6% w/w) and subjected 5 times to ultrasonic sound (Sonorex RK 106, Bandelin, Germany) for 30 s each time, yielding a visually clear dispersion. PDMS films of a mass of 20–100 mg were placed in a 50 mL or 100 mL Erlenmeyer flask and covered with gold particle dispersions, whereat the mass of the dispersion amounted roughly to 20 times the mass of PDMS. The systems were allowed to stand for the periods indicated in the text (typically 7 days). Thereafter the films were removed from the suspensions and dried at ambient conditions. Gold contents in the films (see text) were analyzed with atomic absorption spectroscopy (see above).

## 4. Conclusions

Dichroism of nanocomposites has previously only been described in the visible wavelength region. However, the experiments described above show that dichroism can also be shifted into the near infrared region (NIR). This is evidenced by examples of crosslinked poly(dimethylsiloxane) (PDMS) and gold nanoparticles with a surface layer of 1-dodecanethiol or *tert*-tetradecanethiol. Accordingly, dichroism in the NIR region is accomplished with a sequence of preparation steps, involving diffusion of dispersed particles into swollen crosslinked PDMS films, subsequent drying, exposure of the films to solvent, stretching by a factor of 3–4 in wet state, drying again and annealing. The absorption maximum at φ = 0° is extremely broad and is located between 1500 nm and 2200 nm, while at φ = 90° the absorption maximum arises in the vicinity of the vis-NIR transition. Further, the vis-NIR spectra are characterized by a distinct isosbestic point. The dichroism in the NIR region indicates the presence of oriented linear gold particle assemblies of high aspect ratio, oriented with the long axis parallel to the stretching direction.
